# Polyamidoamine Dendrimers for Enhanced Solubility of Small Molecules and Other Desirable Properties for Site Specific Delivery: Insights from Experimental and Computational Studies

**DOI:** 10.3390/molecules23061419

**Published:** 2018-06-12

**Authors:** Daniel M. Shadrack, Hulda S. Swai, Joan J. E. Munissi, Egid B. Mubofu, Stephen S. Nyandoro

**Affiliations:** 1 Department of Health & Biomedical Sciences, School of Life Science and Bioengineering, The Nelson Mandela Africa Institute of Science and Technology, P.O. Box 447, Arusha, Tanzania; dmssjut@gmail.com (D.M.S.); hulda.swai@nm-aist.ac.tz (H.S.S.); 2 Department of Chemistry, Faculty of Natural and Applied Sciences, St. John’s University of Tanzania P.O. Box 47, Dodoma, Tanzania; 3 Chemistry Department, College of Natural and Applied Sciences, University of Dar es Salaam, P.O. Box 35061, Dar es Salaam, Tanzania; joan.munissi@udsm.ac.tz; 4 Office of the Vice Chancellor, University of Dodoma, P.O. Box 259, Dodoma, Tanzania; ebmubofu@gmail.com

**Keywords:** PAMAM dendrimer, solubility enhancement, drug delivery, small molecules

## Abstract

Clinical applications of many small molecules are limited due to poor solubility and lack of controlled release besides lack of other desirable properties. Experimental and computational studies have reported on the therapeutic potential of polyamidoamine (PAMAM) dendrimers as solubility enhancers in pre-clinical and clinical settings. Besides formulation strategies, factors such as pH, PAMAM dendrimer generation, PAMAM dendrimer concentration, nature of the PAMAM core, special ligand and surface modifications of PAMAM dendrimer have an influence on drug solubility and other recommendable pharmacological properties. This review, therefore, compiles the recently reported applications of PAMAM dendrimers in pre-clinical and clinical uses as enhancers of solubility and other desirable properties such as sustained and controlled release, bioavailability, bio-distribution, toxicity reduction or enhancement, and targeted delivery of small molecules with emphasis on cancer treatment.

## 1. Introduction

In order for a drug to produce a desired therapeutic effect, it is required to reach the targeted site in the right amount and dosage. However, the biomedical potential of many small molecules in clinical utility is limited by their intrinsic poor aqueous solubility [1,2,3]. Solubility is an important parameter required to achieve the concentration of drugs needed for systemic circulation to produce the desired therapeutic effects. Poor solubility results in poor absorption of drugs in the gastrointestinal tract which often results in toxicity [4]. In many pharmaceutical companies, formulation and development of new chemical entities (NCE) or generic drugs are practically limited by poor water solubility, with more than 40% of NCE being insoluble [5,6]. Many approaches to increase solubility and bioavailability of many small molecules in pre-clinical and clinical testing have been reported. One prominent method has been drug complexation with macromolecules such as dendrimers. Since the discovery of dendrimers in 1978 by Fritz Vogtle and in the early 1980s by Donald Tomalia [7,8], polyamidoamine (PAMAM) dendrimers have gained significant attention in biomedical applications as solubility enhancers of poorly soluble molecules [2,3]. Though PAMAM dendrimers bear some toxicity, different strategies including change of dendrimer aggregate and electric state have been suggested to reduce their toxicity [9,10,11,12,13,14]. Previous reviews focused mainly on synthesis, general properties, and applications of dendrimers as drug delivery systems [13,15,16,17,18,19,20,21,22,23]. The present review covers the recent applications of PAMAM dendrimers as drug solubilization agents for poorly soluble small molecules and enhancement of other desirable pharmacological properties with emphasis on applications giving emphasis on cancer treatment, with some highlights on anti-inflammatory, antihypertensive, retinal, antifungal, antioxidant, and antiarthritis drugs.

## 2. Experimental Insights on PAMAM-Drug Interactions for Enhanced Solubility and Other Desirable Pharmacological Properties

### 2.1. Effects of PAMAM Dendrimer on Anticancer Drugs

Many flavonoid and polyphenolic compounds are unsuitable for clinical use due to their extremely poor aqueous solubility. Silybin (**1**), is an antihepatotoxic polyphenolic compound isolated from the milk thistle plant and recognized as the main component of silymarin [24]. Like other flavolignans, clinical uses of silybin is limited by its poor water solubility, poor intestinal absorption, as well as low bioavailability [24,25]. Different efforts to increase the solubility of silybin in pre-clinical and clinical trials have been made. Partially pegylated poly(aminoamide) dendrimer generation four (G4) was synthesized and tested to increase the solubility of silybin [26]. When the dendrimer was conjugated with polyethylene glycol (PEG) chains of 0.55 kDa and 2 kDa, pegylation were found to increase the solubility of silybin [26]. Conjugation with a high PEG chain (2 kDa) increased the solubility of silybin 5-fold compare to a lower PEG chain (0.55 kDa). Furthermore, conjugation of a higher PEG chain sustained the release of silybin for a longer time compared to a lower PEG chain. Pegylation was therefore concluded to assist drug complexation by increasing the number of drugs loaded into the dendrimer. Thus, pegylated dendrimer showed a higher loading capacity than non-pegylated dendrimer. Diaz et al. found that a PEG chain of 2 kDa significantly increased the loading capacity, solubility and sustained the release of silybin compare to a lower PEG chain [26]. 

The flavonoid 3,4-difluorobenzylidene diferuloylmethane (CDF, **2**) is a well-known potent anticancer drug but its clinical applications are hindered by its poor aqueous solubility. Efforts to enhance its solubility for treatment of ovarian and cervical cancer have been attempted. Folic acid (FA)-PAMAM conjugate incorporating CDF was synthesized and characterized by spectroscopic techniques [27]. CDF was then loaded to FA-PAMAM conjugate and free PAMAM dendrimer using the equilibrium dialysis method. However, the anticancer activities of targeted folate drug conjugate (FA-PAMAM-CDF) and the non-targeted folate drug conjugate (PAMAM-CDF) were not significant, although targeted FA-PAMAM-CDF showed high anticancer activities on HeLa and SKOV3 cell lines [27]. The high anticancer activities were attributed to high internalization brought by the FA conjugate. FA-PAMAM-CDF drug formulation further increased the solubility of CDF compared to non-targeted PAMAM-CDF drug formulation [27]. Hyaluronic acid (HA) PAMAM dendrimer formulation of CDF (HA-PAMAM-CDF) for CD44 targeted therapy for prostate cancer has also been developed and its cellular uptake in MiaPaCa-2 cancer cell line studied using fluorescence [28]. It was observed that HA-PAMAM-CDF exhibited higher cellular uptake in MiaPaCa-2 cancer cell line compared to non-targeted PAMAM-CDF formulation [28]. Furthermore, the treatment of CD44 receptors that were overexpressed in MiaPaCa-2 and AsPC-1 human pancreatic cancer cell with targeted HA-PAMAM-CDF resulted in a dose-dependent cytotoxicity compared to non-targeted PAMAM-CDF [28]. Inhibition concentration of CDF was enhanced 1.71-fold in the targeted formulation when compared to free CDF and CDF-PAMAM [28]. Indeed, targeted dendrimer drug delivery formulations appear to be more promising than the non-targeted system in the treatment of cancer and other diseases.

Capecitabine (**3**), is an anticancer drug used for the treatment of various cancers. However, its clinical utility is limited by its adverse side effects that include invasion of healthy tissues. Nabavizadeh et al. conjugated and evaluated the targeted release of capecitabine into colorectal cancer in mice model using PAMAM dendrimers [29]. Both free capecitabine and PAMAM-capecitabine drug complex were administered to mice with colorectal cancer. It was found that PAMAM-capecitabine significantly reduced adenocarcinoma growth as compared to free capecitabine [29]. Furthermore, free capecitabine reduced the number of red blood cells (RBC) and platelet count, whereas, PAMAM-capecitabine showed fewer effects on red blood cells [29]. Remarkably, PAMAM dendrimer complex exhibited sustained release of capecitabine and provided targeted delivery to tumor cells compared to free capecitabine [29].

The delivery of paclitaxel (**4**) for the treatment of ovarian cancer using lipid-dendrimer hybrid has been investigated [30]. Paclitaxel is a hydrophobic anticancer drug used for treatment of various cancers. A hybrid lipid-PAMAM loaded paclitaxel was formulated and evaluated for in vitro and in vivo cytotoxic activity on ovarian cancer cell line [30]. The hybrid nanosystem significantly increased paclitaxel activity by 37-fold compared to free paclitaxel [30]. Further, it was noted that the use of lipid-PAMAM hybrid nanosystem significantly increased the solubility of paclitaxel by approximately 465-fold compared to PAMAM alone [30]. The loading ability of lipid-PAMAM hybrid nanosystem significantly increased as the generation of dendrimer increased. Hybrid PAMAM dendrimer G4 had higher loading capacity than lower hybrid generations (G2 and G3) [30]. The use of lipid-PAMAM hybrid nanosystem proved to be a promising approach for delivering hydrophobic drugs.

In order to provide specific and localized delivery of small molecules, recently, a tumor targeted conjugate system based on asymmetric bow-tie PAMAM dendrimer (ABTD) was synthesized [31]. To this system, biotin was conjugated to serve as the tumor targeting molecule to cancer cells. The second generation taxoid, SB-T-1214 (**5**) was loaded to the system yielding ABTD-tumor-targeting conjugate (ABTD-TTC-1). Cytotoxicity selectivity of free and PAMAM drug conjugates was performed on human breast cancer, murine ovarian cancer, and human lung fibroblast cancer cell lines [31]. The taxoid drug conjugate (ABTD-TTC-1) displayed cytotoxicity against ovarian cancer cells (IC50 = 0.66 nM) that was three times higher than the free taxoid (IC50 = 1.89 nM, 5) indicating the efficiency of the delivery system. ABTD-TTC-1 further exhibited a remarkable selectivity to cancer cells being >1000 times higher compared to human normal cells, thereby presenting the potential of ABTD-based tumor-targeted drug-delivery systems [31]. It was concluded that using biotin receptor PAMAM-drug conjugate achieved an excellent tumor targeted drug delivery, and opened the door for designing cancer therapeutic drug delivery systems [31].

Amine terminated PAMAM dendrimer generation 5 (PAMAM-G5) modified selenium nanoparticles have been formulated for dual delivery of cancer therapeutics of small interfering RNA (siRNA) and cisplatin (**6**) [32]. The formulated delivery system (PAMAM-G5-siRNA-cisplatin) significantly enhanced cytotoxicity through induction of apoptosis [32]. The system PAMAM-G5-Se-cisplatin was found to be less reactive than cisplatin alone when reacted with metallothionein (MT) and glutathione (GSH), suggesting that encapsulation of cisplatin in the nanoformulation would effectively hinder detoxification of the cell [32]. In addition, in vivo study on PAMAM-G5-siRNA-cisplatin system enhanced the antitumor effects without causing abnormality in organs [32]. These findings advocate for the need of integrating both selenium and the gene in drug delivery systems. Literature shows the advantages of targeted drug delivery on increasing the concentration of drugs in the patients’ body, which brings the desired therapeutic effect. These strategies reduce toxicity and dosage frequency, prolong release and protect drug interactions with healthy body tissues whilst ensuring specific delivery of drugs to tissues [33,34,35].

In our group, we synthesized an ethylenediaminetetra-acetic acid (EDTA) core fourth generation PAMAM dendrimer. The synthesized PAMAM dendrimer was deployed to encapsulate the flavonoid, tetramethylscutellarein (TMScu, **7**) to study its aqueous solubility enhancement, in vitro release and thermal stability. The stability of the PAMAM-TMScu complex was found to be generally good at 0, 27, and 40 °C. The synthesized PAMAM-G4 was also capable of encapsulating TMScu (**9**) at 77.8 ± 0.69% encapsulation efficiency and increased the solubility of the flavonoid. The improved pharmacological parameters indicate that bioactivity of TMScu would also be enhanced when administered as a complex (PAMAM-TMScu) [2].

A pegylated PAMAM-G5 functionalized with AS1411 anti-nucleolin aptamers for targeted site-specific delivery of camptothecin (CPT, **8**) (Apt-PEG-PAMAM-CPT) has been synthesized [36]. Camptothecin (8), was encapsulated into the synthesized PAMAM-G5 targeting the treatment of colorectal cancer cell overexpressed in nucleolin receptors [36]. Functionalized aptamer (Apt-PEG-PAMAM-CPT) exhibited a higher encapsulation efficiency of camptothecin and sustained the release for 4-days compared to non-functionalized (PEG-PAMAM-CPT) PAMAM dendrimer [36]. Furthermore, Apt-PEG-PAMAM-CPT specifically delivered camptothecin to nucleolin-positive colorectal cancer cells and efficaciously inhibited the growth of nucleolin-positive cancer cells compared to non-functionalized PEG-PAMAM-CPT and free CPT [36]. Table 1 summarizes the effects of PAMAM dendrimer surface group modifications on the improvement of solubility, bioavailability, and targeted delivery of drugs and on other desirable properties.

Doxorubicin (DOX **9**), is a clinically known broad-spectrum anticancer drug. Clinical applications of this drug are being challenged by its systemic toxicity. Zhong et al. investigated the effect of conjugation of DOX to PAMAM-G4-COOH dendrimer targeting treatment of lung metastasis [38]. Conjugated PAMAM-DOX increased the efficiency in treating lung metastasis in mouse models upon pulmonary drug administration [38]. Furthermore, administration of PAMAM-DOX reduced the number of nodules compared to free DOX, suggesting pulmonary administration of DOX conjugated PAMAM to be a facile nanocarrier for treating lung metastasis [38]. Indeed, as described previously, high PAMAM dendrimer generation improves the loading capacity of drugs, however, their syntheses pose time constraints and are complicated when compared to lower generations. Thus, conjugation of special ligands to increase spaces for drug loading is now an active area in drug delivery research. In a particular study aimed to improve the activity of DOX for treatment of cervical cancer, DOX was physically loaded to the PAMAM-G4.5 conjugated with IL-6 antibody and a peptide, arginine-glycine-aspartate (RGD) tripeptide [39]. IL-6 and RGD were conjugated to provide targeted delivery and internalization delivery of DOX to cervical cancer cells. PAMAM-IL6/DOX showed higher encapsulation efficiency of 51.3% when compared to PAMAM-RGD/DOX which had 30.1%. PAMAM dendrimer conjugated with IL6 (PAMAM-IL6-DOX) was found to have higher drug loading and faster release which corresponded to greater cytotoxicity. It was concluded that, IL6-PAMAM conjugate is the best approach for targeted delivery of DOX to cervical cancer cell [39].

Surface groups present on PAMAM dendrimers greatly affect its loading ability and drug incorporation into the cavities of the dendrimer. The terminal/surface groups further have an effect on the solubilization of PAMAM dendrimer. The effects of hydroxyl and amine terminated PAMAM dendrimer generation 5 (PAMAM-NH2 and PAMAM-OH) on increasing solubility, equilibrium dialysis, and loading of an antitumor drug 5-fluorouracil (5-FU, **10**) have been investigated [40]. PAMAM-NH2 showed higher incorporation of a 5-FU drug by three times compared to PAMAM-OH terminated dendrimer, whereby 70% and 14% of 5-FU (**10**) drugs were incorporated into PAMAM-NH2 and PAMAM-OH, respectively. The decrease of entropy (S < 0) favored higher incorporation of the drug into PAMAM-NH2 than for PAMAM-OH demonstrating high entropy (S > 0) of the system [40]. Furthermore, PAMAM-NH2 increased the solubility of 5-FU three times compared to PAMAM-OH. In addition, it was observed that weakly acidic drugs like 5-FU bind weakly to hydroxyl terminated PAMAM dendrimers, as 5-FU cannot protonate the hydroxyl groups of PAMAM-OH unlike PAMAM-NH2 [40]. Despite the high solubility and incorporation effects that PAMAM-NH2 has over PAMAM-OH on 5-FU, the former is still challenged by toxicity [54]. Thus, in order to reduce the toxicity of PAMAM-NH2, surface modification by pegylation or combined drug delivery strategies are very much required.

Alkaloids are an important group of compounds with potent biological activities but their poor pharmacokinetic (PK) profiles have greatly contributed to their limited exploitations in clinical applications. For instance, berberine (**11**) is a potent anticancer alkaloid. However, poor PK properties have restricted berberine from many pre-clinical and clinical applications. The release of berberine in an animal model and in vitro settings was recently investigated [41]. Berberine was conjugated and encapsulated to fourth generation PAMAM dendrimer. Conjugation achieved high drug loads at 37.49% when compared to encapsulated berberine which had 29.9% drug loading. In vitro release was carried out in water and phosphate buffer saline 7.4 (PBS 7.4) media for both conjugated and encapsulated berberine. In PBS 7.4 media, berberine was highly released up to 80% and 98 % of the encapsulated and conjugated berberine, respectively whereas, the release in water was 72% and 98 %, respectively. Anticancer activities of both formulations were tested against breast cancer cell lines MCF-7 and MDA-MB-468, where both formulations showed higher activities compared to free berberine. However, PAMAM-berberine drug conjugate showed significantly higher activity than encapsulated berberine [41]. Moreover, the PK of PAMAM-berberine formulation and the free was investigated in an albino rat model, parameters such as half-life, initial concentration, and elimination rate constant were studied. PAMAM-berberine conjugate showed interesting PK properties with a half-life of 14.33 h compared to 6.7 h for free berberine. PAMAM simultaneously increased the pharmacokinetic profile of berberine with low hemolytic toxicity which suggested good biocompatibility. PAMAM-berberine conjugation was suggested as the best drug delivery option rather than encapsulation [41].

Efforts to improve targeted drug delivery to the tumor and simultaneously increase solubility and bioavailability of a poorly soluble drug, paclitaxel (PTX, **4**), for treatment of upper gastric cancer (UGC) have been reported. It is now contended that conjugation of drugs with fatty acids not only provides enhanced targeted drug delivery to the tumor but also enhances anticancer activities, as fatty acids are highly taken up by a tumor. For instance, omega-3 fatty acid, docosahexaenoic acid (DHA), is readily taken up by tumor cells, and hence, provides reduced tumor growth [42]. Thus, in order to enhance the anticancer activities of paclitaxel, DHA was conjugated to the synthesized amine terminated PAMAM-G4 dendrimer to provide a PAMAM-G4-DHA-PTX and PAMAM-G4-PTX drug conjugates [37]. The anticancer activities of PAMAM-G4-PTX and PAMAM-G4-DHA-PTX were investigated in vitro [37]. It was observed that PAMAM-G4-DHA-PTX significantly induced cell deaths in UGC treatment compared to PTX alone or PAMAM-G4-PTX conjugate. These findings suggested that conjugation of omega-3 fatty acids to PAMAM dendrimer incorporating PTX is a promising strategy for improving its activities and targeted delivery.

Gallic acid (**12**) is a natural compound with known antioxidant and anticancer activities, however, its clinical uses are disadvantaged due to poor bioavailability. Efforts to increase the bioavailability of gallic acid have been attempted by various research groups. Abduo and Masoud evaluated bioavailability of gallic acid by conjugating it to PAMAM dendrimer and phosphatidylcholine (PC) [42]. Both systems, PAMAM and PC sustained the release of gallic acid for several hours compared to the free one. Furthermore, gallic-PAMAM and gallic-PC conjugate improved bioavailability, provided hepatoprotection thereby improving the histological appearance of liver cells [42]. Conjugation of gallic acid to PAMAM or PC thus provided a means to improve its bioavailability

Bendamustine (**13**) is an anticancer agent belonging to the class of nitrogen mustard compounds inducing cell death by apoptosis [43]. Bendamustine is unstable and can readily be hydrolyzed to two non-active compounds [43]. Attempts to improve its stability using various nanomedicine approaches have been attempted. Gothwal et al., formulated a drug delivery system based on PAMAM-G4 and conjugated bendamustine. PAMAM improved the stability of bendamustine and sustained release for up to 72 h with improved activity on leukemia cells compared to free bendamustine [43]. Interestingly, PAMAM increased the concentration of bendamustine to the tumor cells and improved pharmacokinetic properties in vivo. PAMAM dendrimers–bendamustine showed more effectiveness in delivering bendamustine than free bendamustine [43].

**Figure 1:**
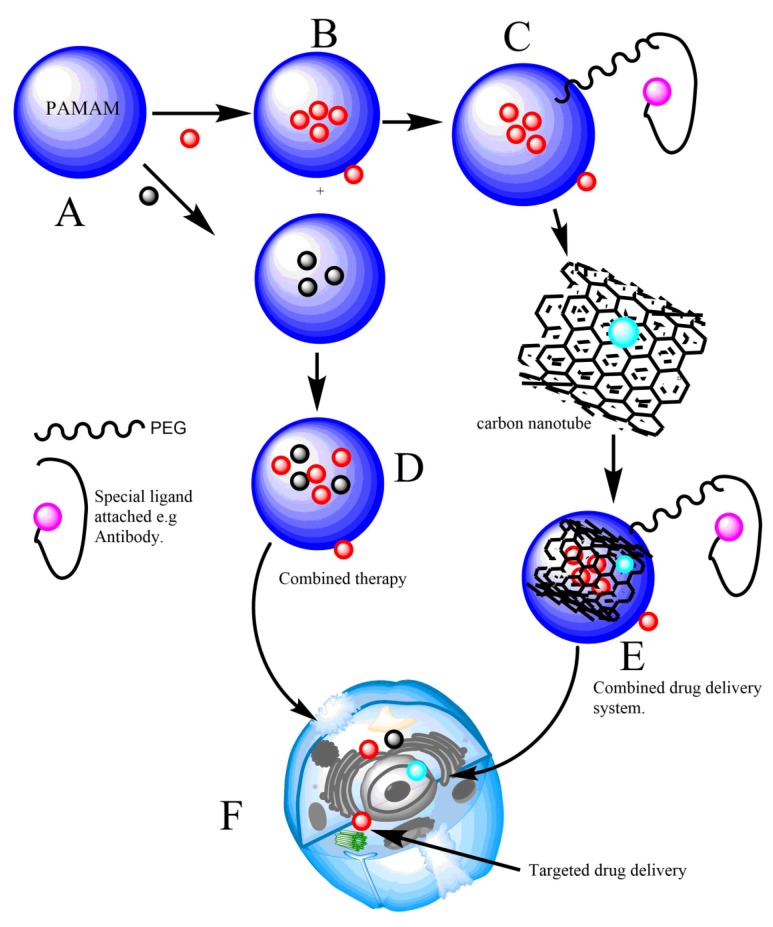
**A** scheme representing two systems which is promising in improving the solubility of poorly water soluble molecules. **B**,**D** show a combined therapy drug delivery system for improved solubility, in such a system, two different drugs are loaded into the dendrimer separately, then the two nanosystems are combined together to have one system with two different drugs. The two drugs are both delivered to cell **F**. **C**,**E** represent a combined drug delivery system, in such a system, two different nanoparticles are prepared separately loaded or unloaded with drugs. Then, the two systems are combined together and drug is released. Conjugation with polyethylene glycol PEG and/or special ligands such as antibody to polyamidoamine (PAMAM) dendrimer nanohybrid not only improves solubility and sustains release of drugs but also enhances targeted drug delivery. Red, black, and blue balls represent drugs entrapped or conjugated into the nanohybrid system.

Xu et al. designed a multifunctional pH-sensitive self-fluorescent drug delivery system based on mesoporous silica nanoparticle and PAMAM dendrimers [44]. The system showed interesting properties including high reactive sites, retaining drug molecules, controlling drug delivery behavior as well as imaging properties [44]. The system was investigated for its release mechanism by using curcumin (**14**) whereby the drug was loaded into the modified PAMAM–mesoporous silica nanoparticle (PAMAM-MSN). In the release studies of curcumin (**14**) from MSN and PAMAM-MSN, the former showed high release rate, while, the latter sustained the release of curcumin for longer hours at the pH conditions under study. It was further observed that the percentage release of curcumin decreased as the PAMAM generation increased, despite having a higher loading capacity than MSN [44]. These findings concluded that the PAMAM-MSN could serve as a drug delivery and imaging system. PAMAM-mesoporous silica nanoparticle is a good example of PAMAM nanohybrid described in Figure 1.

Yesil-Celiktas et al. formulated a mesoporous silica–PAMAM as a novel drug delivery system for black carrot anthocyanins (BCA, **15**) [45]. The BCA, (**15**) was encapsulated into silica–PAMAM and the release mechanism and biological activity enhancement were investigated on the proliferative effects of neuroblastoma (Neuro 2A). It was observed that silica–PAMAM sustained the release of BCA for six days and enhanced the anti-proliferative activity on Neuro 2A [45]. Further, the free silica–PAMAM was observed to be less toxic to cells [45]. Capping of silica to PAMAM dendrimer significantly reduced the cytotoxicity of PAMAM dendrimer, suggesting the silica–PAMAM dendrimer is an effective drug delivery system.

### 2.2. Effects of PAMAM Dendrimer on Anti-Inflammatory Drugs

Solubility enhancement of different generations of PAMAM dendrimer to anti-inflammatory molecules that are in pre-clinical and clinical trials have been intensively investigated. Naproxen (NAP, **16**), is a non-steroidal anti-inflammatory drug (NSAID) of the propionic acid class that serves as a nonselective COX inhibitor in the treatment of pain, fever, and stiffness. However, its clinical uses are hampered by poor solubility. Najlah et al. investigated the effect of PAMAM dendrimer generation and functionalization on solubility enhancement of NAP [46]. NAP was conjugated to PAMAM dendrimers of lower generation (G0) in a ratio of 1:1 and high generations (G3) in different ratios using a diethylene glycol linker. Both generations were functionalized using a lauroyl chain. It was found that at lower pH non-modified PAMAM dendrimer generation G0 and G3 showed significant solubility enhancement of NAP to counterpart lauroyl chain functionalized PAMAM dendrimer generation. Moreover, conjugated NAP to PAMAM dendrimers showed high inhibition concentration on Caco-2 cells compared to free NAP. It was further found that higher PAMAM dendrimers (G3) functionalized with lauroyl chain had high toxicity compared to lower generation (G0). Furthermore, conjugated NAP had high permeability to Caco-2 cells monolayer compared to free NAP. This suggested that lauroyl chain should be functionalized to lower PAMAM generation (G0) rather than high PAMAM generations for optimal drug delivery and solubility of NAP. Similar observations were previously reported by Patel et al. and Choudhary et al. for solubility enhancement studies of various PAMAM dendrimer generations to aceclofenac (**17**), another poorly soluble NSAID drug [55,56].

### 2.3. Effects of PAMAM Dendrimers on Antihypertensive Drugs

Combination therapy (Figure 1 has now become one of the best options in treatment and combating many diseases including cancer and problems associated with multi-drug resistance compared to mono-therapy. For instance, in cancer treatment, combined therapy has brought several advantages over monotherapy such as reduced number of stem cells, reduction in metastasis and tumor growth as well as induction of apoptosis [57]. Similarly, in the treatment of cardiovascular diseases, the use of combined therapy has been highly recommended [58]. In order to achieve the goal of combined therapy in hypertension treatment, for instance, the ability of PAMAM dendrimer to load and deliver two antihypertensive drugs; ramipril (RAPL, **18**) and hydrochlorothiazide (HCTZ, **19**) were investigated [47]. A non-linear solubility relationship between the two loaded drugs was observed at different pHs, PAMAM concentration and PAMAM surface groups. For instance, RAPL (**18**) showed high solubility improvement with 4.91-fold in amine terminated PAMAM, while HCTZ (**19**) showed the highest solubility improvement with 3.72-fold in carboxyl terminated PAMAM dendrimer. It was further noted that PAMAM-RAPL and PAMAM-HCTZ showed higher dissolution rate than the free drugs. In a similar way, PAMAM-hybrid i.e. PAMAM-RAPL/HCTZ showed higher dissolution rate than individual PAMAM-drug complexes. Physicochemical studies on PAMAM-hybrid showed stability in a dark and cooled environment, suggesting it as an innovative strategy for combined therapy not only in hypertension but also in other diseases such as cancer [47]. 

Bioavailability and solubility enhancement of the drug, candesartan cilexetil (CC, **20**), were studied using PAMAM dendrimers of different generations and terminal functional groups i.e. COOH, NH2, and tris (hydroxymethyl) aminomethane (TRIS) [59]. Solubilization effect of PAMAM to CC (**20**) was found to be a function of PAMAM dendrimer concentrations and generations. It was further observed that anionic PAMAM dendrimer functionalized with COOH group and the neutral PAMAM dendrimer functionalized with TRIS enhanced the solubility of CC compared to amine terminated PAMAM dendrimers. Generally, anionic PAMAM dendrimers are suggested to be more ideal for drug delivery than cationic NH2 ending surface group dendrimers due to their positive surface charges [48,59]. Furthermore, anionic PAMAM dendrimers ending with COOH are commonly observed to be less toxic than cationic PAMAM-NH2 terminated dendrimers [48]

### 2.4. Effects of PAMAM Dendrimer on Delivering Retinal Drugs

Trials on how to improve the treatment of eye diseases have prompted the use of PAMAM dendrimers for delivering drugs to the retina. Thus, the use of PAMAM dendrimers on the release of retinal drugs has been investigated. For example, the effect of dendrimer generations on the loading and release of dexamethasone (DEX, **21**) for treatment of diabetic retinopathy has been investigated using Sprague–Dawley rats to determine the release effects of DEX from PAMAM dendrimer to the retina [49]. The release of DEX was observed to be slow for G3 PAMAM dendrimer for both in vitro and in vivo tests [49]. Furthermore, it was observed that cell viability was low for G3-PAMAM-DEX complex. Thus, PAMAM dendrimer increased the concentration of DEX (**21**) to both cornea and sclera tissues compard to free DEX [49].

In the treatment of blind disorders, achieving high-level transgene expression without causing adverse side effects has remained a serious drawback in retinal treatment. To address such a drawback, a hydroxyl terminated PAMAM dendrimer functionalized with amine groups for delivering triamcinolone acetonide (TA, **22**) to human retinal pigment epithelium was synthesized [50]. The dendrimer was further conjugated with PEG to improve the stability of the designed system thereby achieving effective treatment with minimal effect on transgene delivery [50].

### 2.5. Effects of PAMAM Dendrimer on Antifungal Drugs

Many antifungal drugs also suffer from clinical uses hampered by their poor solubility profiles. Several efforts to increase solubility of many antifungal drugs have been carried out over the past years, for example, cyclodextrin has previously been extensively studied as solubility enhancer for many antifungal drugs [60,61] An attempt to improve the solubility of an antifungal drug, amphotericin B (**23**), whose clinical use has been hampered by poor water solubility and nephrotoxicity, was done using PAMAM dendrimer [51]. Amphotericin B (**23**), was loaded to PAMAM dendrimer of different generations [51]. Solubility enhancement of amphotericin B was studied in vitro. It was observed that the solubility effect of amphotericin B was a function of an increase in dendrimer concentration and generation [51]. Moreover, dendrimer generation and high concentration sustained the release of the drug. The developed formulation was found to be stable in the dark and at cooler temperature [51].

### 2.6. Effects of PAMAM Dendrimer on Antioxidant Compounds

Combined drug delivery approaches have demonstrated interesting properties in improving solubility and bioavailability of poorly soluble drugs compared to the single drug delivery system approach. The former has an ability to improve solubility as well as cellular uptake of drugs compared to the latter. Probuco (PB, **24**) is a sparingly water-soluble drug known to possess antioxidant, anti-inflammatory, and hypocholesterolemic properties [52]. In order to improve the solubility and bioavailability of probucol (**24**), a combined drug delivery system composed of pegylated PAMAM dendrimer (PEG-PAMAM-G5/G7) and nanostructured lipid carrier (NLC) was designed [52]. PB-NLC was formulated and then pegylated PEG-PAMAM-G5/G7 incorporated to form PEG-PAMAM-G5/PB-NLC and PEG-PAMAM-G7/PB-NLC. PB-NLC/PEG-PAMAM-G5/G7 was observed to significantly improve PB solubility. It was further noted that the combined drug delivery system, PEG-PAMAM-G7/PB-NLC sustained the release and improved the stability of PB compared to PB-NLC. High plasma concentration, oral bioavailability, and the cholesterol lowering effects of PB were highly achieved in the combined system as compared to free or PB-NLC complex [52]. A combined drug delivery approach, therefore presents a suitable drug delivery platform for sparingly water soluble and oral bioavailability of drugs compared to a single drug delivery approach. Therefore, an adaptation of this approach will pave the way to enhance the bioactivities of compounds whose clinical utility is limited by poor water solubility and oral bioavailability

Resveratrol (**25**), is one of the stilbene compounds known to be effective as anti-ageing and antioxidant agents. However, resveratrol suffers from poor water solubility. Several formulation attempts to improve its solubility have been conducted. Some formulations have been taken aboard for commercialization. Pentek et al., developed a resveratrol formulation based on PAMAM dendrimer to enhance its solubility, transdermal penetration, and stability. It was shown that PAMAM-G4 improved solubility, stability, and skin penetration of resveratrol. PAMAM-G4 successfully loaded resveratrol provide a new direction in formulation and commercialization of products based on PAMAM-G4 [53].

### 2.7. Effects of PAMAM Dendrimer on Antiarthritis Drugs

Intra-articular delivery of drugs by injection for treatment of osteoarthritis, a common degenerative disease of the articular joints, is challenged by rapid clearance of drugs from the joint cavity. Thus, controlled release of drugs to the chondrocytes for long retention of drugs has been sought as a solution for the treatment of osteoarthritis. In order to achieve sustained release of drugs to chondrocytes, Hu et al., developed a targeted drug delivery system based on pegylated PAMAM dendrimer to deliver drugs to articular cartilage [62]. Two PAMAM conjugates were synthesized i.e. targeted and non-targeted (PEG-PAMAM). The former carrier was conjugated with chondrocyte affinity peptide (CAP), to form CAP-PEG-PAMAM nanocarrier. In vitro and in vivo studies on the developed targeted system showed better targeting specificity and cellular uptake in chondrocytes. Furthermore, the system showed no toxic effects suggesting good biocompatibility [62]. The developed CAP-PEG-PAMAM was therefore suggested as a novel approach that could deliver small molecules specifically to chondrocytes.

**Figure 2:**
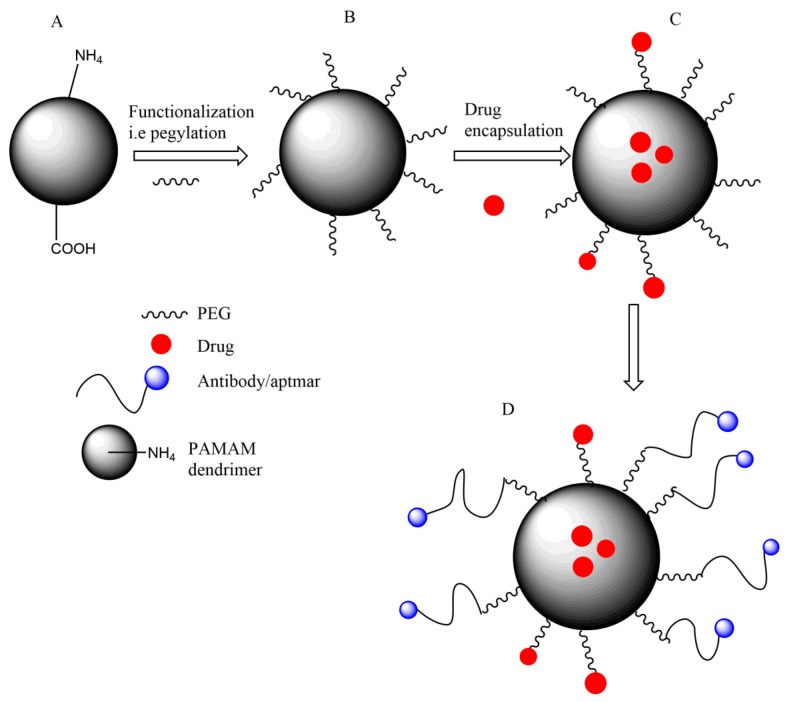
**A** cartoon scheme showing functionalization and conjugation to PAMAM dendrimer. PAMAM dendrimer can be functionalized by pegylation as indicated in **B**, normally PEG is used to modify the surface of the PAMAM dendrimer. Functionalized PAMAM dendrimer can be used to encapsulate drugs to improve solubility and biodistribution as shown in **C**. Drugs into PAMAM dendrimer can be encapsulated into the interior core of the dendrimer or can be conjugated into the surface of the dendrimer. **D** shows that functionalized PAMAM dendrimer can further be conjugated with special ligands such as antibody/aptamer for improved targeted drug delivery.

**Figure 3:**
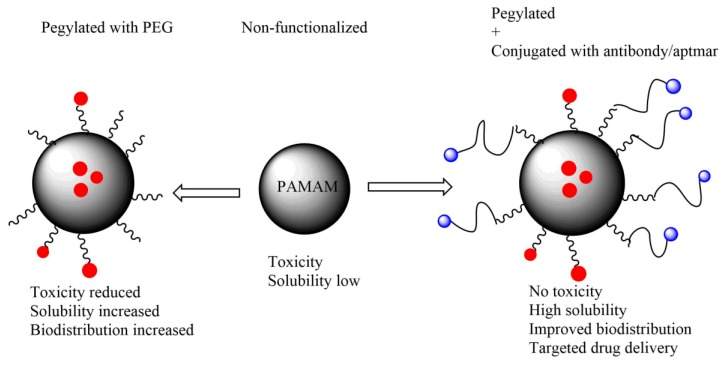
A cartoon showing the difference between non-functionalization PAMAM dendrimer, pegylated and pegylated PAMAM dendrimer conjugated with antibody/aptamer. Non-modified PAMAM dendrimer shows considerable toxicity when compared to functionalized. Pegylated PAMAM dendrimer shows improved solubility and biodistribution of encapsulated drugs, the antibody/aptamer conjugated PAMAM dendrimer has an added advantage of targeted drug delivery. Pegylated and conjugated PAMAM dendrimers represents a better drug delivery approach.

**Figure 4:**
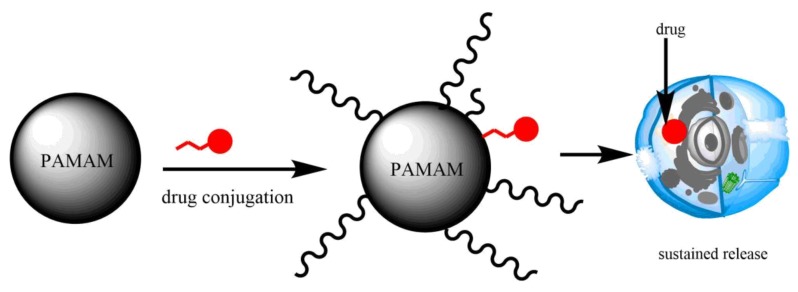
A representation of PAMAM dendrimer drug conjugate. Dendrimers can conjugate drugs at the surface prior or after being functionalized. Conjugated drugs are often released for a longer time than encapsulated drugs.

Generally, non-functionalized PAMAM dendrimers have demonstrated lower cytotoxicity than some surface functionalized PAMAM dendrimers. Several groups have worked on a number of strategies to reduce the resulting toxicity. Such strategies include conversion of an ester terminal to a carboxylic acid ending group [48]. Furthermore, anionic PAMAM dendrimers have been shown to be less toxic than cationic PAMAM dendrimers on different cells [54,63]. Surface modification of PAMAM dendrimer (Figure 1, Figure 2, Figure 3 and Figure 4 not only reduces the toxicity but also increases the solubility of the encapsulated/conjugated drugs and their biodistribution. Functionalized PAMAM dendrimer conjugated with special ligands such as antibody shows great efficiency by having targeted drug delivery to tumor cells due to increased drug loading [32,36,45,54]. Figure 2 indicates the properties of non-functionalized and functionalized PAMAM dendrimers with PEG and/or special ligands which improve its function.

## 3. Computational Insight of PAMAM-Drug Interactions for Improved Solubility

### 3.1. PAMAM Dendrimer-Drug Interaction with Anti-TB Drugs

The use of computational studies in drug design is a growing field today. Developed and available algorithms have assisted in providing insight into molecular, atomistic and mechanistic interaction of small molecules with macromolecules. Recently, the interactions between PAMAM dendrimers with drugs for enhancing bioactivities of small molecules were investigated using both experimental and computational methods. Molecular dynamics and molecular docking atomistic simulation approaches have been employed in studying PAMAM dendrimer drug interactions, and, have provided useful information. For example, the interaction of rifampicin (RIF, **26**), an anti TB drug with fourth PAMAM dendrimer generation was investigated by using molecular dynamics simulation and experimental methods [64]. RIF was loaded into PAMAM-G4 by a docking approach and the stability of the complex investigated at neutral and acidic pH. Twenty molecules of RIF were loaded into PAMAM-G4 dendrimers and subjected to 100 ns MD simulation for stability studies. It was observed that at neutral pH, RIF-PAMAM complex was relatively stable compared to low pH, where RIF was rapidly released to the bulk surface of the dendrimer [64]. These findings were further supported by other experimental findings which demonstrated that at low pH, the release of drugs is triggered more than at neutral pH [2]

### 3.2. PAMAM Dendrimer-Drug Interaction with Anticancer Drugs

The effect of dendrimer generations, charge, counter-ions, and structured water on the dynamic interaction of 5-FU, (**10**) drug with peptide based dendrimer were investigated using a molecular dynamics simulation [65]. Molecular dynamics simulation was carried out at constant number of particles, volume, and temperature (NVT) and pressure (NPT) ensembles and the system was solvated with a TIP3P water model [65]. CHARMM and CGENFF general force fields were used for peptide dendrimer and 5-FU, respectively, and all calculations were performed using NAMD [65]. It was observed that peptide-dendrimer carrier did not show long-time retention of 5-FU (**10**) within the hydrophobic core. Instead, 5-FU interacted with all dendrimer parts with hydrogen bonds being formed. It was further found that charges on the periphery of the dendrimer have a negative effect on 5-FU drug-carrier interactions [65]. However, 5-FU interacted effectively with neutral dendrimer, compared to charged dendrimers. It was concluded that the presence of structural water and solvated counter-ions on the periphery of the dendrimer inhibited 5-FU dendrimer interaction. However, charges played a great role in the prevention of agglomeration of the dendrimer. The neutral dendrimer of higher generation increased the 5-FU drug interaction [65].

A molecular dynamic simulation study was employed to investigate the binding effects of four anticancer drugs on pegylated and non-pegylated PAMAM dendrimer [66]. The four anticancer drugs; doxorubicin (DOX, **8**), methotrexate (MTX, **27**), chlorin e6 (**28**), and 7-ethyl-10-hydroxycampothecin (SN38, **29**) were encapsulated into pegylated and non-pegylated G3-PAMAM dendrimer. The simulation was carried out at constant NPT and NVT ensembles in vacuum and solvent [66]. It was observed that PAMAM dendrimer showed strong binding to the four drugs. The effect of pegylation was observed on drug loading as well, pegylated PAMAM dendrimer accommodated more drugs than non-pegylated dendrimers. The study supported the importance of functionalization for improving drug loading, as PEG showed weak intramolecular interaction with drugs, but still assisted in entrapping more drug to PAMAM dendrimer where they bound very tightly compared to in PEG chains [66].

**Figure 5:**
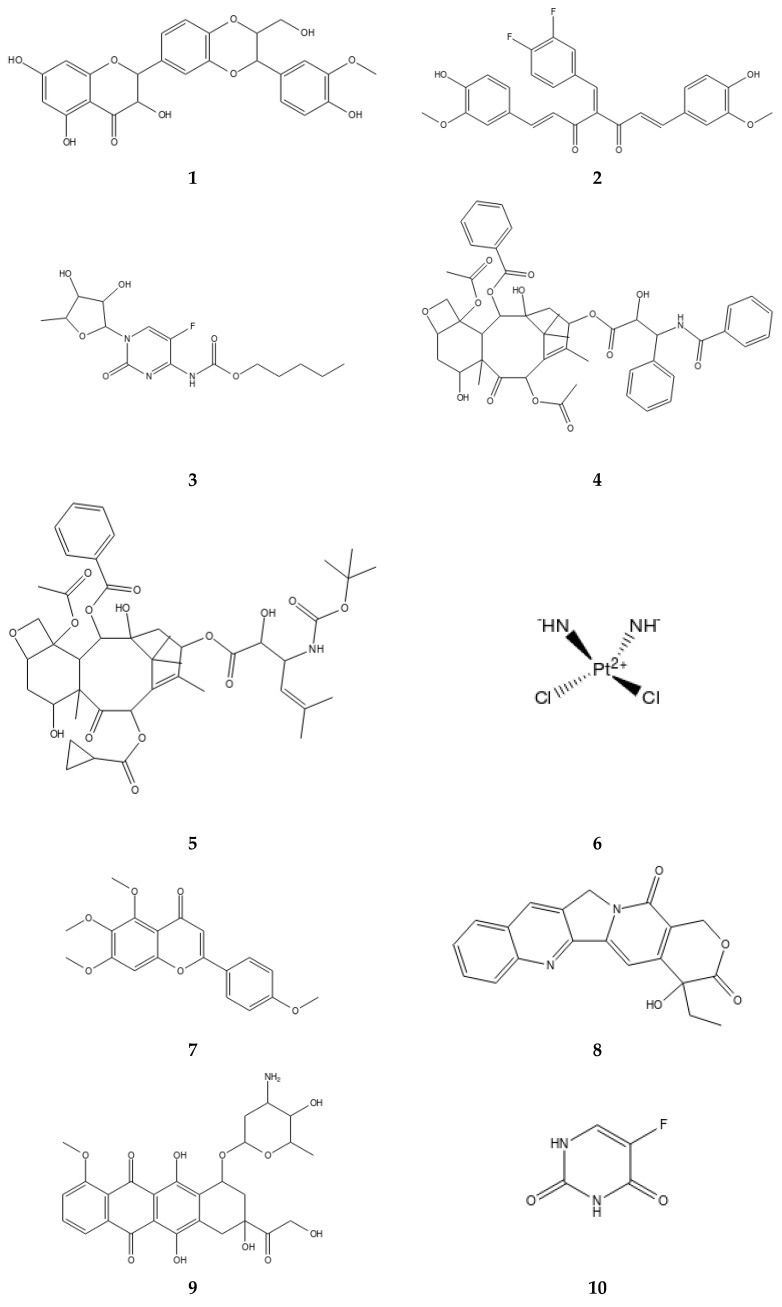

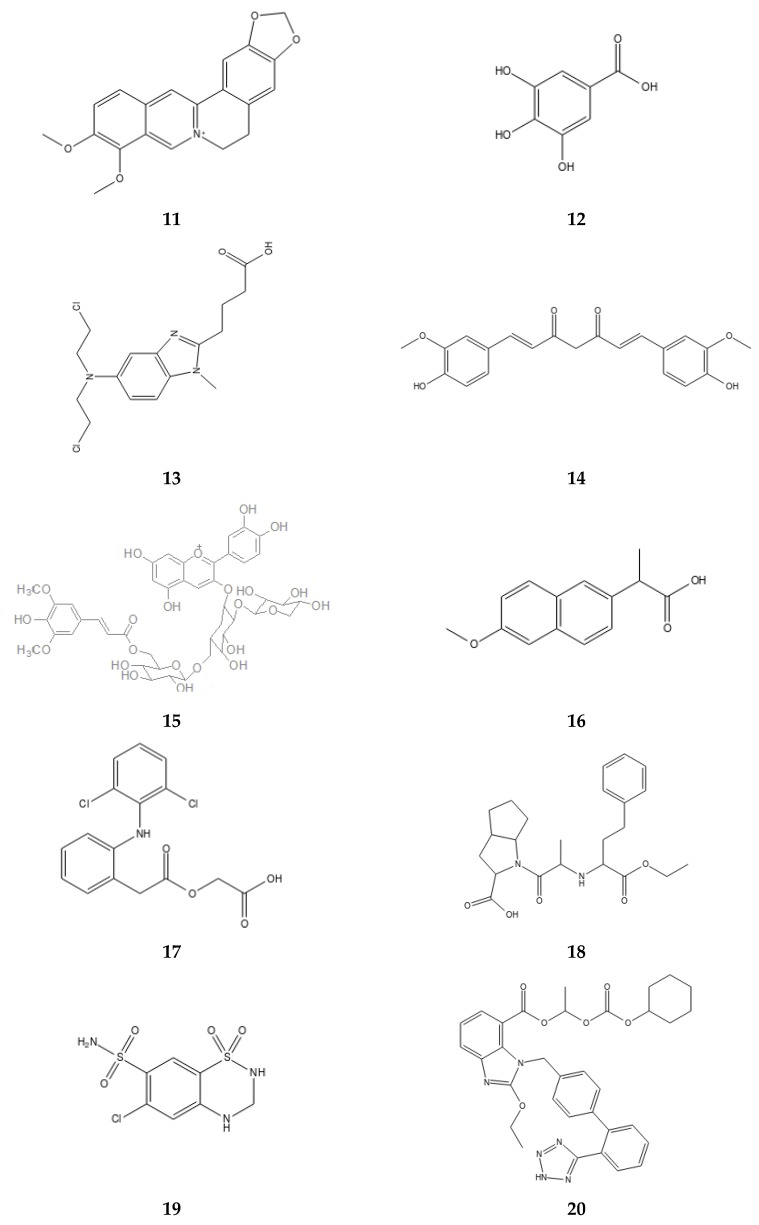

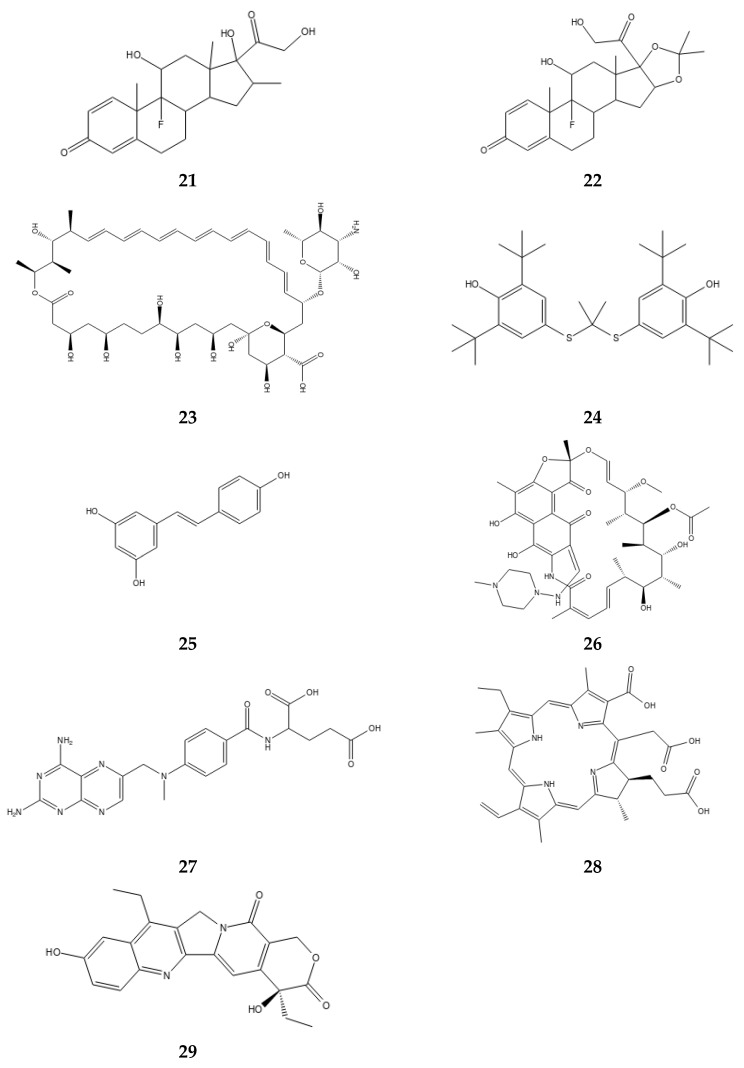
Some of the small molecules of which PAMAM dendrimer has enhanced their solubility and other desirable properties for site specific delivery in pre-clinical and clinical trials. These molecules were investigated using experimental and/or computational approaches.

The interaction of PAMAM dendrimer generations with 5-FU (**10**) drug was explored using a combination of experimental and in silico methods [67]. 5-FU (**10**) was encapsulated to PAMAM dendrimer generations 0.5–2.5 by molecular docking [67]. High dendrimer generations formed a stable complex with 5-FU drugs. Biophysical studies on PAMAM dendrimer-5FU drug interaction showed that PAMAM-5FU sustained the release of 5-FU, and a low percentage i.e. less than 8% of 5-FU was released from PAMAM as compared to 16% of free 5-FU [67]. Molecular docking further provided an insight into the mechanism of 5-FU binding into PAMAM dendrimer generations. Indeed, computational study, although not extensively carried out to explore the interactions of the PAMAM-drug complex for drug solubilization, a few existing reports have paved the way on how PAMAM dendrimer interacts with drugs. Figure 5 shows some of the drugs in pre-clinical and clinical trials in which PAMAM dendrimer have enhanced their solubility and other desirable pharmacological properties. 

## 4. Conclusions

The article reviewed recent applications of PAMAM dendrimers in biomedical applications with special emphasis on drug solubility. Different techniques used in PAMAM-dendrimer drug formulation which significantly enhanced controlled release, targeted delivery and other desirable pharmacological features of small molecules with more focus on anticancer agents were discussed. Surface modification of PAMAM dendrimers was noted to significantly enhance and increase solubility, drug loading, and sustained release of drugs. Furthermore, conjugation of PAMAM with special ligands such as antibody provided a targeted drug delivery approach, a promising approach in tumor or cancer treatment. Combined drug delivery systems not only increased solubility and bioavailability of drugs but also sustained the release of small molecules compared to a single delivery system. Along with the formulation strategies, factors such as pH, PAMAM dendrimer generation, PAMAM dendrimer concentration, nature of the PAMAM core, the surface of PAMAM dendrimer, and the nature of the drug encapsulated have profound influences on the solubilization effects of PAMAM dendrimer. Some PAMAM dendrimer-drug formulations have been commercialized, rasveratrol serving as a notable example. In addition, some patents exist which claim the potential application of PAMAM dendrimers in delivering drugs such as anticancer drugs and antibiotics. Current and future investigations on the use of PAMAM dendrimers for solubilization of molecules should consider the co-application of combined therapy and a combined drug delivery system to form a single PAMAM nanohybrid system for significantly enhancing the clinical application of small molecules whose utility is limited by poor solubility in the body. 

## Figures and Tables

**Table 1:** Some examples on the effect of polyamidoamine (PAMAM) dendrimer surface modification on improvement of solubility, biodistribution, and targeted drug delivery of small molecules.

PAMAM Formulation	Drug Loaded	Formulation Type	Effects Observed/Results	References
PEG-PAMAM-G4	Silybin (1)	Encapsulation	High PEG increased solubility	[26]
FA-PAMAM	CDF (2)	Encapsulation	Increased solubility and targeted delivery of CDF	[27]
HA-PAMAM	CDF (2)	Encapsulation	Increased cellular uptake & reduced toxicity	[28]
PAMAM	Capecitabine (3)	Conjugation	Targeted delivery to tumor & less toxicity to cell	[29]
PAMAM-lipid hybrid	Paclitaxel (4)	Encapsulation	Increased solubility & activity	[30]
PAMAM-Biotin	SB-T-1214 (5)	Conjugation	High potency & targeted drug delivery	[31]
PAMAM-G4-DHA	Paclitaxel (6)	Conjugation	Increased activity in UGC treatment	[37]
G5-PAMAM-NH2-Se/siRNA	Cisplatin (6)	Encapsulation	Enhanced toxicity	[32]
G4-PAMAM-NH2	TMScu (7)	Encapsultion	Increased solubility	[2]
G5-PAMAM-NH2-aptamers	CPT (8)	Encapsulation	Sustained release	[36]
Apt-PEG-PAMAM	CPT (8)	Encapsulation	Targeted drug delivery	[36]
G4-PAMAM-COOH	DOX (9)	Conjugation	Efficiency in treating lung metastasis	[38]
IL6-PAMAM-G4.5	DOX (9)	Conjugation	Targeted delivery of DOX to cervical cancer cells	[39]
PAMAM-COOH/NH2	5-FU (10)	Encapsulation	Archieved high loading & low toxicity	[40]
PAMAM-NH2	5-FU (10)	Encapsulation	Improved solubility & bonding	[40]
PAMAM	Berberine (11)	Conjugation & Encapsulation	Improved pharmacokinetic profile	[41]
PAMAM	Gallic acid (12)	Conjugation	Improved bioavailability	[42]
PAMAM-G4	Bendamustine (13)	Conjugation	Improved stability and pharmacokinetics	[43]
PAMAM-MSN	curcumin (14)	Encapsulation	Sustained release of curcumin for long time	[44]
Silica-PAMAM	BCA (15)	Encapsulation	Sustained release, less toxicity & enhanced activity	[45]
PAMAM-G0-lauroyl	NAP (16)	Conjugation	Increased solubility & low toxicity	[46]
PAMAM-hybrid	RAPL (18) & HCTZ (19)	Encapsulation	High solubility improvement	[47]
PAMAM-NH2/COOH/TRIS	Candesartan cilexetil (CC) (20)	Conjugation	anionic dendrimer enhanced solubility	[48]
PAMAM-G3	DEX (21)	Encapsulation	Controlled release	[49]
PEG-PAMAM-NH2	Triamcinole acetonide (TA) (22)	Conjugation	High level transgene expression	[50]
PAMAM	Amphotercin B (23)	Encapsulation	Increased solubility	[51]
PEG-PAMAM-G7 or 5/NLC	PB (24)	Encapsulation	Improved solubility & oral bioavailability	[52]
PEG-PAMAM-G5/7-NLC	Probuco (PB) (24)	Encapsulation	High oral bioavailability and activity	[52]
PAMAM-G4	Rasveratrol (25)	Encapsulation	Improved solubility succeded for commercialization	[53]
